# Match running performance profiles of amputee football players at the national level

**DOI:** 10.1038/s41598-023-36856-0

**Published:** 2023-06-19

**Authors:** Jarosław Muracki, Michał Nowak, Adam Kawczyński, Ana Filipa Silva, Filipe Manuel Clemente

**Affiliations:** 1 Institute of Physical Culture Sciences, Faculty of Health and Physical Education, University of Szczecin, 70-453 Szczecin, Poland; 2 Collegium Medicum Dr.Władysław Biegański, Department of Physical Culture Sciences, Jan Długosz University in Częstochowa, 42-200 Częstochowa, Poland; 3 Gdańsk University of Physical Education and Sport, Poland, 80-336 Gdańsk, Poland; 4 Instituto Politécnico de Viana do Castelo, Escola Superior Desporto e Lazer, Rua Escola Industrial e Comercial de Nun’Álvares, 4900-347 Viana do Castelo, Portugal; 5 Research Center in Sports Performance, Recreation, Innovation and Technology (SPRINT), 4960-320 Melgaço, Portugal; 6 Research Centre in Sport Sciences, Health Sciences and Human Development (CIDESD), Quinta de Prados, Edifício Ciências de Desporto, 5001-801 Vila Real, Portugal; 7 Instituto de Telecomunicações, Delegação da Covilhã, 1049-001 Lisbon, Portugal

**Keywords:** Physiology, Risk factors, Musculoskeletal system

## Abstract

Even though running performance and positional profiles in football are well described, amputee football (AF) has different characteristics of the movement, pitch dimensions, and time played. There is a gap in the scientific literature about positional profiles based on running performance in AF. This study aimed to investigate the differences between positions, the influence of the amputation level or defect of the lower limb (LD), the differences in running performance between halves, and the relationship with the final match result. Thirteen AF National Team players were monitored by Global Navigation Satellite System (GNSS), tracking 24 official international matches for 17 months. Values of top speed, peak acceleration, peak deceleration, average distance per minute, sprint mean speed, GPS load per minute, inertial load per minute (Gs load/min), number of sprints per minute, and a number of impacts per minute were analyzed for defenders (DEF), midfielders (MID) and forwards (FOR). Additional factors analyzed were amputation level (below the knee, low amputation—LA or over the knee, high amputation—HA or defect of the lower limb—LD) and the match's final result. Midfielders had significantly higher running performance parameters compared to other positions (greater top speed than DEF (+ 0.3 m/s; *p <* 0.001) and FOR (+ 0.2 m/s; *p =* 0.045), greater peak acceleration and deceleration than DEF (+ 0.5 m/s^2^ for both measures; *p <* 0.001) and FOR (+ 0.4 and + 0.3 m/s^2^; *p <* 0.001 and *p =* 0.036, respectively), greater GPS load/minute than DEF (+ 0.2 load/min; *p =* 0.001) and FOR (+ 0.3 load/min; *p <* 0.001), greater Gs load per minute than DEF (+ 2.7 load/min; *p <* 0.001) and FOR (+ 1.8 load/min; *p <* 0.001), greater number of impacts per minute than DEF (+ 0.2 n/min; *p <* 0.001) and FOR (+ 0.2 n/min; *p <* 0.001). Players with LD had significantly higher running performance than those with LA or HA. In the match's second half, a decrease in running performance was registered. The trend of running more when losing could be observed—AF players had higher running parameters in lost matches, but the differences were not statistically significant. Further research complied with contextual game analysis is needed to assess the running performance of AF players deeply.

## Introduction

Amputee football (AF) is a team ball sport gaining popularity in different countries^1^. AF is a variation of football in which field players (FP) have amputations or defects of the lower limb and goalkeepers (GK) in the upper limbs. The AF team consists of six FP and a GK on the pitch playing against the opposite team in a 2 × 25 min match. The FP use lofstrand crutches to move around the pitch. They cannot use artificial leg prosthesis nor touch the ball with the crutches^1,2^. In modern team ball, sports training control and regulation is a methodological procedure to optimize adaptation and maximize the match performance of the players^3–6^. Progress in the player's performance is reached by the training process based on the interplay of external and internal loads imposed on players, which are implemented according to loads observed in championship matches^7^. The match requirements in football are described in great detail in the scientific literature, and a lot of scientific data is available. In contrast, the scientific literature about AF is scarce^2^. Therefore, there is a gap in the scientific literature with the lack of reliable information about the match loads occurring in AF and describing the game demands in different positions: defenders (DEF), midfielders (MID), and forwards (FOR)^8^. Following the methods used in able-bodied football, this kind of reliable information is necessary for coaches and conditioning specialists to design training programs specific for playing position considering the level of amputation^9^ and control the training process as well to have a point of reference for performance monitoring of AF matches^10^.

The scientific literature about AF is relatively scarce, and their results cannot be directly compared. Often research on AF contains a minimal number of players and analyzes a limited number of matches or both, like in Esatbeyoglu et al.^11^ study. The results of the pilot study of Nowak *et al.*^12^ monitoring only four games is hard to compare to the other studies because of the shorter of 10 min match time and including GK in the analysis, which radically decreases the presented averages. In the study of Maehana et al.^10^ Japanese AF players were analyzed, but neither sport level nor type of rivalry was clearly specified. Their results showed, in general lower total distances for the high-level amputation group compared to low-level amputation group^10^. The pilot study of Muracki *et al.*^8^ aimed to examine, with a well-established methodology, the running performance and pain experienced by the AF field players at the national level and to identify further research directions. Another obstacle to directly comparing results from different articles is the other reference ranges of speed zones used. Therefore, it is essential to standardize the GPS tracking methodology to increase the replicability and ensure the possibility of research comparing, considering the population and context of each study^13^. Moreover, all the above-mentioned AF studies had a small experimental group and a small number of matches analyzed as a limitation.

Knowledge about match load helps to manage the team better and plan the training process in case of its effectiveness, but another question connected with the load is the prediction of injury risk. Injury prevention in disabled sports is an important issue^14–16^. Still, the lack of scientific evidence about the load and demands of AF matches causes difficulties in planning the training and injury prevention process^8^.

Considering the above-identified gaps in scientific literature, the need for practical applications based on scientific evidence for trainers and medical staff working in AF, and the limitations of yet-published studies, the present study aimed to determine the match running performance and the profile of different FP positions in AF. Also, it aimed to describe the differences in key performance indicators (KPI) between the first and second half of a match with long-period monitoring of top-level AF players during international competitions. Finally, the contextual aspect (as the final game result), the level of amputation, and the defect of the lower limb were also investigated.

## Materials and methods

### Participants

Previously twenty players were involved in the research, although 3 of them were excluded from the analysis because of being goalkeepers, two were excluded by the coach because of the AF rules limiting the size of the team, and finally, the next two suffered injuries which excluded them from participation. Finally, thirteen adult male AF players aged 30.8 ± 8.0 years, with 176.8 ± 7.0 (cm) of height and a body mass of 74.2 ± 7.8 (kg), were involved in the study. All participants are representatives of the Polish National Amputee Football Team. Eligibility criteria were: (i) a presence and active participation in the matches during international tournaments, Euro Championship, and World Cup; (ii) no illness, no injuries; and (iii) no taking any medicine. This study included only FP (7 DEF, 3 MID, and 3 FOR). GK’s were excluded from the study due to the different characteristics of this AF position, i.e. having two functional lower limbs and a significant limitation of the field on which GK can move during the match. There were eight players with lower limb amputations (2 above the knee—HA, 6 under knee—LA) and 5 players with different defects (congenital agenesia) of the lower limb (LD) resulting in abbreviation, deformation and limb not being fully functional which is in accordance with official rules of the AF^17,18^. Seven of the FP have healthy right lower limb, and the other six have healthy left lower limb. Only four players used crutches in everyday life, and the others used crutches only for playing and training AF. The average years of playing AF was 6.8 ± 2.7 years (Table 1).

**Table 1:** Group characteristics.

Parameter	All players (n = 13)	Defenders (n = 7)	Midfielders (n = 3)	Forwards (n = 3)
Stature (cm)	176.8 ± 7.0	176.4 ± 5.6	173.3 ± 9.7	181.3 ± 7.6
Body mass (kg)	74.2 ± 7.8	73.9 ± 7.6	70.3 ± 10.0	78.8 ± 6.2
Age (years)	30.8 ± 8.0	32.6 ± 7.3	25.7 ± 6.7	32.0 ± 11.5
Training experience years (years)	6.8 ± 2.7	6.1 ± 3.1	8.0 ± 2.0	7.0 ± 3.0
Disability type	Amputation: 8	Amputation: 5	Amputation: 0	Amputation: 3
	Defect of the lower limb: 5	Defect of the lower limb: 2	Defect of the lower limb: 3	Defect of the lower limb: 0
Amputation height	Transfemoral: 2	Transfemoral: 1	Transfemoral: n.a	Transfemoral: 1
	Transtibial: 6	Transtibial: 4	Transtibial: n.a	Transtibial: 2
Healthy limb	Right lower limb: 7	Right lower limb: 3	Right lower limb: 2	Right lower limb: 2
	Left lower limb: 6	Left lower limb: 4	Left lower limb: 1	Left lower limb: 1
Using LC everyday (%)	30.8% (4 out of 13)	28.6% (2 out of 7)	33.3% (1 out of 3)	33.3% (1 out of 3)

Legend: LC – Lofstrand Crutches.

### Study overview

**Figure 1:**
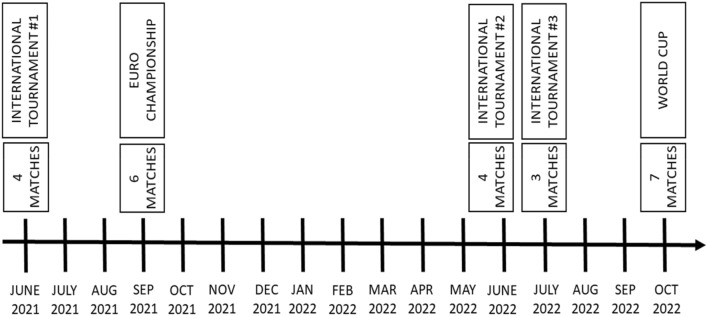
Timeline of the events and number of matches. The time–space gap between international matches is caused mainly by the Covid-19 pandemic.

This exploratory study observed one cohort of 13 AF players from Polish National Amputee Football Representation for 17 months (from June 2021 to October 2022). Through the monitoring period, the players attended five international tournament events playing 24 official matches. Only international competitions of the highest level were included in the study to ensure the highest quality of the game and conditions. Every match of these events was monitored using the GNSS system, delivering several parameters (Fig. 1).

### GNSS tracking methods and conditions

The research used a GNSS sensor from Integrated Bionics Inc., 2020 (USA) called TITAN2 (technical parameters are provided in Appendix B). The data was initially prepared using the manufacturer's web platform (export to Excel) and then subjected to statistical analysis. All device and software settings were original and were not modified for this research.

To determine the actual workload of key performance indicators (KPIs) using GNSS technology, an analysis of 24 matches during international tournament events was carried out. A similar methodology was maintained in all games, keeping the manufacturer's cold-start recommendations. The total monitoring time was about 120 min: 15 (min) cold start of GNSS devices, 15 (min) control of the correct operation of the devices and putting them into the players' vests, 30 (min) warm-up, 2 × 25 (min) of play with a 10 (min) break plus eventual timeouts and additional time. Furthermore, each player wore the same device in every match to reduce inter-unit error. After extracting data from the devices, the clod start, control time, warm up and break were excluded. The study only considers the actual playing time of individual players on the pitch, including all timeouts requested by the coach and additional time if they occurred. After rejecting data of poor quality (e.g. moving sensor, other factors interrupting the connection, playing time shorter than 5 min), 293 tracking data files were obtained (120 of defenders, 89 of midfielders and 84 of forwards). The average playing time was 23 (min) ± 8.1. These data were then put into more detailed and statistical analysis, delivering a number of parameters.

To characterize the external load number of parameters were registered: (i) distance per minute (m/min); (ii) session time (min); (iii) activity time (min); (iv) peak acceleration (m/s^2^); (v) peak deceleration (m/s^2^); (vi) top speed (m/s); (vii) mean sprint distance (m); (viii) mean sprint speed (m/s); (ix) number of sprints per minute (n/min); (x) the number of impacts per minute (n/min); (xi) GPS load per minute (load/min); and (xii) inertial load per minute (Gs load/min). A description of all parameters can be found in Appendix A.

In addition to the sampling frequency of the device (10 Hz), positioning measurement accuracy and its derivatives are influenced by other factors. The most important include the number of satellites connected and their horizontal dilution of precision (HDOP)^19,20^. HDOP values > 1 are defined as ideal, and values > 2 as excellent^21^. In this study, the average number of satellites was 17.8 ± 2.9 and HDOP 1.46 ± 0.34.

### Ethics

The experiments reported in the manuscript were performed in accordance with the ethical standards of the Helsinki Declaration. Subjects participated in the study of their own free will and were informed that they could terminate their participation at any time. Every participant read and signed an informed consent form. The project of this study was approved by the Senate Research Ethics Committee of the University School of Physical Education in Wroclaw, Poland (project identification code: 26/2016 approval date: 13.10.2016).

### Statistical procedures

A priori sample size estimation was conducted using G*power software (version 3.1, University of Dusseldorf, Germany). A recommended sample size of 15 participants was estimated for three groups, assuming a fixed effect size of 1.2 (large), a power of 0.95, and a p-value of 0.05. The descriptive statistics are presented in the form of mean and standard deviation. The normality and homogeneity of the sample were explored and analyzed using Kolmogorov–Smirnov and Levene tests. After confirmation of normality by the assumption of the Central Limit Theorem (n > 30) and homogeneity of the sample (*p* > 0.05), the one-way ANOVA was conducted to compare the outcomes of playing positions, type of injury/amputation, and final score. Eta squared was used to analyze the effect size. A post hoc test was applied while using the Tukey test. The paired t-test was used to compare the variations of outcomes between halves. The Cohen d test was executed to analyze the pairwise effect size. The statistical procedures were performed in the SPSS software (version 27.0.0.0., IBM, Chicago, USA) for a *p <* 0.05.

## Results

After excluding tracks shorter than 5 min of session time from all collected data of 24 matches, a total of 293 tracks were analyzed (Table 2).

**Table 2:** The number of tracks, session time, activity time and its ratio for players of different positions (mean ± SD).

Parameter	All players (n = 13)	Defenders (n = 7)	Midfielders (n = 3)	Forwards (n = 3)
Number of registered tracks (n)	293	120	89	84
Average session time (min)	23.0 ± 8.1	22.7 ± 8.3	25.6 ± 6.5	20.6 ± 8.5
Average activity time (min)	11.6 ± 4.4	11.5 ± 4.3	14.0 ± 4.1	9.1 ± 3.6
Activity time to session time ratio (%)	50.4	50.7	54.7	44.2

Session time is the period of time when a player was on the pitch during a match. Activity time is the period of time in which a player was moving on the pitch.

Table 3 shows the running parameters and mechanical measures per playing position. One-way ANOVA revealed significant differences between playing positions for top speed (*p <* 0.001), peak acceleration (*p <* 0.001), peak deceleration (*p <* 0.001), sprint mean speed (*p =* 0.017), distance per minute (*p <* 0.001), GPS load per minute (*p <* 0.001), Gs load per minute (*p <* 0.001), and impacts per minute (*p <* 0.001).

**Table 3:** Descriptive statistics (mean ± SD) of top speed, acceleration, and deceleration and standardized locomotor and mechanical measures per playing position.

	Defenders	Midfielders	Forwards	One way ANOVA
Top speed (m/s)	5.8 ± 0.8^$^	6.1 ± 0.7^#,¶^	5.9 ± 0.6^$^	F = 7.488; *p <* 0.001; $${\eta }^{2}$$=0.047
Peak acceleration (m/s^2^)	3.3 ± 0.5^$^	3.8 ± 0.4^#,¶^	3.4 ± 0.5^$^	F = 26.485; *p <* 0.001; $${\eta }^{2}$$=0.149
Peak deceleration (m/s^2^)	3.7 ± 0.8^$^	4.2 ± 0.7^#,¶^	3.9 ± 0.6^$^	F = 9.710; *p <* 0.001; $${\eta }^{2}$$=0.060
Sprint mean speed (m/s)	4.7 ± 0.2	4.7 ± 0.2^¶^	4.7 ± 0.2^$^	F = 4.173; *p =* 0.017; $${\eta }^{2}$$=0.038
Average distance per minute (m/min)	66.9 ± 11.7^¶^	70.2 ± 9.0^¶^	58.9 ± 10.6^#,$^	F = 26.628; *p <* 0.001; $${\eta }^{2}$$=0.149
GPS load (load/min)	0.7 ± 0.4^$,¶^	0.9 ± 0.3^#,¶^	0.6 ± 0.2^#,$^	F = 15.229; *p <* 0.001; $${\eta }^{2}$$=0.091
Gs load (load/min)	4.8 ± 2.1^$,¶^	7.5 ± 2.2^#,¶^	5.7 ± 2.4^#,$^	F = 39.382; *p <* 0.001; $${\eta }^{2}$$=0.207
Sprints (n/min)	0.1 ± 0.1	0.1 ± 0.1	0.1 ± 0.1	F = 1.454; *p =* 0.236; $${\eta }^{2}$$=0.014
Impacts (n/min)	0.2 ± 0.2^$^	0.4 ± 0.3^#,¶^	0.2 ± 0.1^$^	F = 40.415; *p <* 0.001; $${\eta }^{2}$$=0.243

Significantly different from ^#^ Defender; ^$^ Midfielder; ^¶^ Forward for a *p <* 0.05.

Tukey post hoc test showed that MID achieved a significantly greater top speed than DEF (+ 0.3(m/s); *p <* 0.001) and FOR (+ 0.2(m/s); *p =* 0.045). Moreover, MID achieved significantly greater peak acceleration and deceleration than DEF (+ 0.5(m/s^2^) for both measures; *p <* 0.001) and FOR (+ 0.4 and + 0.3(m/s^2^); *p <* 0.001 and *p =* 0.036, respectively). Midfielders also achieved a significantly greater sprint mean speed than FOR (+ 0.09(m/s); *p =* 0.024).

Forwards covered a significantly smaller distance per minute than MID (–11.3 (m/min); *p <* 0.001) and than DEF (–8.0(m/min); *p <* 0.001). Midfielders had a significantly greater GPS load per minute than DEF (+ 0.2(load/min); *p =* 0.001) and FOR (+ 0.3 (load/min); *p <* 0.001). Similarly, MID had significantly greater Gs load per minute than DEF (+ 2.7 (load/min); *p <* 0.001) and FOR (+ 1.8 (load/min); *p <* 0.001). Finally, MID presented a significantly greater number of impacts per minute than DEF (+ 0.2 (n/min); *p <* 0.001) and FOR (+ 0.2 (n/min); *p <* 0.001).

Table 4 shows the variations of top speed, acceleration, and deceleration and standardized locomotor and mechanical measures per type of amputation or limb defect. One-way ANOVA revealed significant differences between amputation type for top speed (*p =* 0.002), peak acceleration (*p <* 0.001), peak deceleration (*p =* 0.002), sprint mean distance (*p =* 0.024), sprint mean speed (*p =* 0.010), distance per minute (*p <* 0.001), GPS load per minute (*p <* 0.001), Gs load per minute (*p <* 0.001), and impacts per minute (*p <* 0.001).

**Table 4:** Descriptive statistics (mean ± SD) of top speed, acceleration, and deceleration and standardized locomotor and mechanical measures per type of amputation.

	Low (under the knee)	Defect leg	High (over the knee)	One way ANOVA
Top speed (m/s)	5.9 ± 0.8^$¶^	6.1 ± 0.6^#,¶^	5.7 ± 0.4^#,$^	F = 6.458; *p =* 0.002; $${\eta }^{2}$$=0.043
Peak acceleration (m/s^2^)	3.5 ± 0.5^$,¶^	3.7 ± 0.4^#,¶^	3.2 ± 0.4^#,$^	F = 12.422; *p <* 0.001; $${\eta }^{2}$$=0.079
Peak deceleration (m/s^2^)	3.9 ± 0.8^$,¶^	4.1 ± 0.7^#,¶^	3.6 ± 0.5^#,$^	F = 6.415; *p =* 0.002; $${\eta }^{2}$$=0.042
Sprint mean distance (m/min)	18.1 ± 4.7	19.2 ± 5.1^¶^	15.9 ± 4.9^$^	F = 3.778; *p =* 0.024; $${\eta }^{2}$$=0.035
Sprint mean speed (m/s)	4.7 ± 0.2^¶, $^	4.7 ± 0.2^#,¶^	4.6 ± 0.1^$^	F = 4.729; *p =* 0.010; $${\eta }^{2}$$=0.043
Average distance per minute (m/min)	60.2 ± 10.7^$,¶^	71.2 ± 9.3^#,¶^	65.4 ± 9.1^#,$^	F = 40.707; *p <* 0.001; $${\eta }^{2}$$=0.219
GPS load (load/min)	0.6 ± 0.3^$,¶^	0.9 ± 0.3^#,¶^	0.6 ± 0.2^#,$^	F = 24.611; *p <* 0.001; $${\eta }^{2}$$=0.145
Gs load (load/min)	4.3 ± 1.8^$,¶^	6.8 ± 2.3^#,¶^	7.8 ± 1.6^#,$^	F = 69.334; *p <* 0.001; $${\eta }^{2}$$=0.324
Sprints (n/min)	0.1 ± 0.1	0.1 ± 0.1	0.1 ± 0.1	F = 1.482; *p =* 0.230; $${\eta }^{2}$$=0.014
Impacts (n/min)	0.2 ± 0.2^$,¶^	0.4 ± 0.3^#,¶^	0.2 ± 0.1^#,$^	F = 22.217; *p <* 0.001; $${\eta }^{2}$$=0.152

Significantly different from ^#^ Low; ^$^ Defect; ^¶^ High for a *p <* 0.05.

Players with defective leg showed significantly greater top speeds than those with low (+ 0.2 (m/s); *p =* 0.016) and high amputations (0.4(m/s); *p =* 0.008). Players with DL had significantly greater peak acceleration and deceleration than players with HA (+ 0.4 and + 0.5(m/s); *p <* 0.001 and *p =* 0.005, respectively). Sprint mean distance was significantly greater in players with DL than in players with HA (3.4(m/min); *p =* 0.029). Distance covered per minute was significantly greater in players with DL than with LA (+ 11.1(m/min); *p =* 0.026) or HA (+ 5.8(m/min); *p =* 0.011). GPS load per minute was significantly greater in players with DL than with LA (+ 0.25(load/min); *p <* 0.001) or HA (+ 0.28(load/min); *p <* 0.001). On the other hand, Gs load per minute was significantly greater in players with HA than in players with LA (+ 3.5(load/min); *p <* 0.001) or with DL (+ 1.0(load/min); *p =* 0.040). The number of impacts was significantly greater in players with DL than in players with LA (+ 0.2(n/min); *p <* 0.001) and HA (+ 0.2(n/min); *p =* 0.001).

Table 5 shows the variations of top speed, acceleration, deceleration, and standardized locomotor and mechanical measures per final score. One-way ANOVA revealed significant differences between the final score for top speed (*p =* 0.037).

**Table 5:** Descriptive statistics (mean ± standard deviation) of top speed, acceleration, and deceleration and standardized locomotor and mechanical measures per final score.

	Lost	Draw	Win	One way ANOVA
Top speed (m/s)	6.1 ± 0.6	6.1 ± 0.7	5.8 ± 0.8	F = 3.326; *p =* 0.037; ${\eta }^{2}$=0.022
Peak acceleration (m/s^2^)	3.6 ± 0.5	3.6 ± 0.4	3.5 ± 0.5	F = 1.084; *p =* 0.340; ${\eta }^{2}$=0.007
Peak deceleration (m/s^2^)	4.0 ± 0.7	4.1 ± 0.9	3.8 ± 0.8	F = 2.998; *p =* 0.051; ${\eta }^{2}$=0.019
Sprint mean speed (m/s)	4.7 ± 0.2	4.7 ± 0.2	4.7 ± 0.2	F = 1.296; *p =* 0.276; ${\eta }^{2}$=0.012
Average distance per minute (m/min)	66.1 ± 10.7	61.5 ± 8.5	65.9 ± 11.9	F = 1.483; *p =* 0.228; ${\eta }^{2}$=0.01
GPS load (load/min)	0.8 ± 0.3	0.7 ± 0.3	0.7 ± 0.3	F = 0.715; *p =* 0.490; ${\eta }^{2}$=0.005
Gs load (load/min)	5.7 ± 2.3	5.0 ± 2.1	6.0 ± 2.4	F = 1.785; *p =* 0.170; ${\eta }^{2}$=0.012
Sprints (n/min)	0.1 ± 0.1	0.1 ± 0.1	0.1 ± 0.1	F = 0.595; *p =* 0.552; ${\eta }^{2}$=0.006
Impacts (n/min)	0.2 ± 0.2	0.2 ± 0.2	0.3 ± 0.3	F = 1.254; *p =* 0.287; ${\eta }^{2}$=0.010

**Figure 2:**
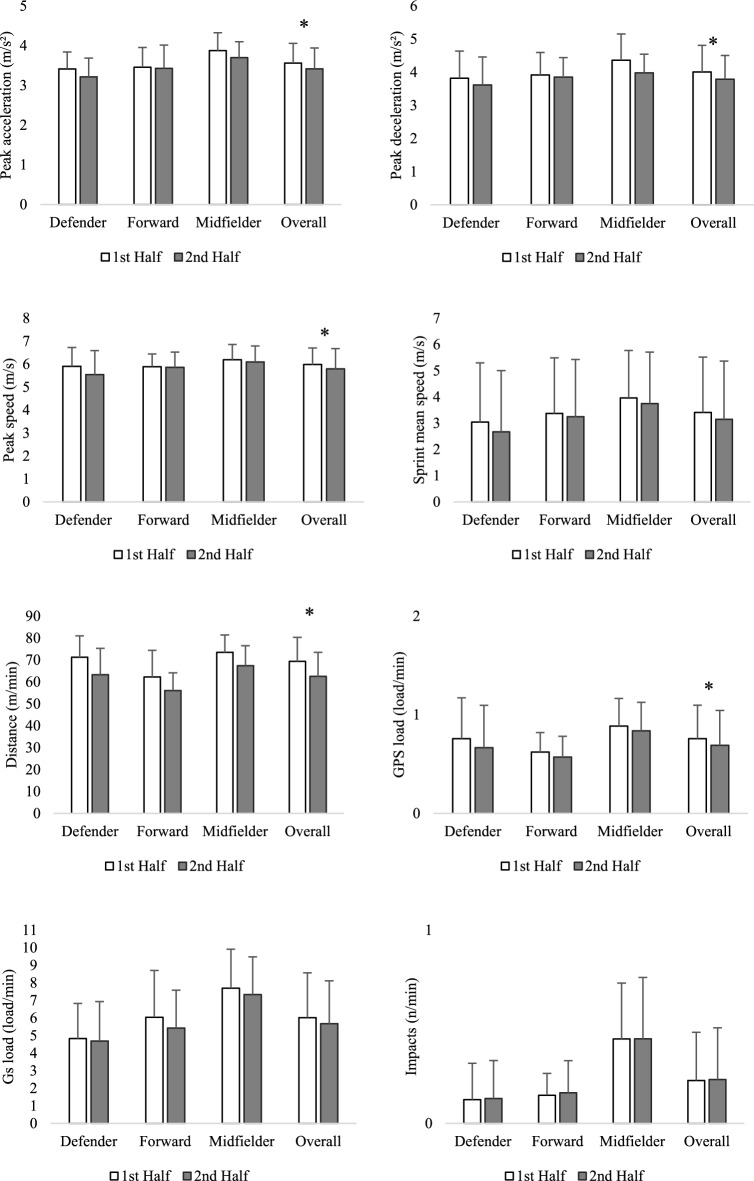
Descriptive statistics (mean and standard deviation) of locomotor and mechanical outcomes between halves. The paired-t-test was performed only for the overall group, so * marks differences as statistically significant only for comparing the 1st and 2nd halves.

Figure 2 shows the descriptive statistics of the locomotor and mechanical outcomes between halves. Paired t-test comparing locomotor and mechanical measures between halves revealed a significant decrease in performance in the second half in the following outcomes: top speed (–0.18(m/s); t = 2.062; *p =* 0.041; d = 0.246); peak acceleration (–0.16(m/s^2^); t = 2.777; *p =* 0.006; d = 0.317); peak deceleration (–0.19(m/s^2^); t = 2.168; *p =* 0.032; d = 0.254); distance per minute (–7.2(m/min); t = 6.258; *p <* 0.001; d = 0.668); GPS load per minute (–0.09(load/min); t = 2.209; *p =* 0.029; d = 0.476). No significant differences between halves were found in sprint mean speed (– 0.04(m/s); t = 1.307; *p =* 0.196; d = 0.199), Gs load per minute (– 0.37(load/min); t = 1.424; *p =* 0.157; d = 0.148) and impacts (+ 0.02(load/min); t = 0.663; *p =* 0.509; d = 0.086).

## Discussion

So far, to the author's best knowledge, there has yet to be any study of such a long-term nature of observation as in the present study. The works of Nowak AM et al. (2020)^12^ and Maehana et al. (2022)^10^ gave some contradictory information due to the difference in the methodology and the equipment used. And above all, they are based mainly on the total distance, which in the current sports practice reality is no longer sufficient^22–24^. The current global trend in football research is performance analysis in relation to the context of the game^22,23,25–27^. Staying in line with this idea, our study was the first step to describing the positional profiles of the AF players. Our research shows differences in performance between positions (Table 2) which makes it possible to determine the positional profiles^23^. The highest top speed, average running distance per minute, highest values of accelerations, decelerations and highest number of impacts per minute characterize the profile of a midfielder. Complex parameters like GPS load per minute and Gs load per minute from the accelerometer are also the highest for MID. The defender's profile has the least dynamic characteristics—parameters of top speed, accelerations, decelerations and Gs load per minute are the lowest and statistically significantly lower compared with MID. The profile of forwards stands out from the rest because of the lowest distance covered per minute and lowest GPS load per minute (statistically significant differences compared to the two other positions). Currently, there are no materials in the literature that can be compared with our results in AF, but they are similar to the corresponding positions in the football^22,24,28^.

There are also distinct differences relative to amputation level and defect of the lower limb (Table 3). The differences caused by amputation level were previously observed by Maehana et al.^29^, which is in accordance with our results. Players with defects of the lower limb achieve the highest top speed and accelerations and decelerations values. Also, average distance per minute, GPS load per minute and the number of impacts per minute have the highest values for this group of players which means that players of this group run the most and are the most dynamic. In contrast, the group of players with high amputation levels had the lowest values of top speed, accelerations, decelerations (significant differences compared to two other positions), and mean sprint distance (significantly lower than MF). Players with high amputation levels had significantly higher Gs load per minute parameters compared to the two other groups, which the specific biomechanics of their movement may cause. In this study, there were significant differences in running performance between players with a high level of amputation, low level of amputation and defect of the lower limb. Still, it is essential to mention that since there is a relationship between running performance and the position on the pitch, there may be a complex interaction between these factors. However, the level of amputation or having a lower limb defect stays significant in the biomechanics of movement^30,31^. Players with amputations may find an advantage in their previous activity before the amputation, especially if it had been football. Players with lower limb defects may find an advantage in the life-long adaptation to whole-body biomechanics and movement patterns. Maehana *et al.*^10^ came to similar conclusions where the low-level amputation group had significantly higher total distance covered and sprint values than the high-level amputation group^10^.

Analysis of the relationship between the match's final result and running performance shows that the average distance per minute, GPS load, top speed and the number of accelerations were the highest in lost games. The main goal of the losing team is to regain the chance to win or tie as quickly as possible. Our research confirms that such a relationship is visible with a negative or tie result, although it is not statistically significant. In the won matches, the number of impacts and Gs load had the highest values. In contrast, the Gs load was the lowest in draw matches. In draw matches, nearly all parameters are very similar to the lost ones except Gs load and average distance per minute, which are the lowest compared to win or lose. Although there was a visible trend showing that players run more and faster in matches they lose, the differences were not so sharp to be statistically significant. Still, this relationship to be proved or denied needs further studies, including several different teams.

Differences between match halves were observed in the present study, showing a decrease in performance in running parameters (Fig. 2.). Simim *et al.*^32^ presented results showing a decrease between halves in total distance and average distance per minute (same reduction of both parameters: − 11%, − 8% and – 12% for DEF, MID and FOR, respectively) claims that observed differences are small despite the fact that the effect size was ranging from small (0.3 for MID), through moderate (0.5 for DEF) to large (0.9 for FOR)^32^. Currently, total distance and even relative distance are considered as important but insufficient because they do not give precise information without contextual analysis^24,33^. In the present study, we observed a statistically significant decrease in peak acceleration, peak deceleration, peak speed, average distance per minute and GPS load per minute in the second half compared to the first half (Fig. 2.). Reducing the decrease of performance in the second half, especially in the last minutes of the match could bring benefits in effectiveness and advantage over the opponent with possible influence on the final result, other authors of research also confirm this in the field of football^34^.

### Practical application

Knowledge about characteristics of positional profiles in AF enables the opportunity for optimal selection of the players for positions of defenders, midfielders and forwards. Individual profiling of players based on the match requirements for specific positions and the performance indications derived from these data enables the opportunity for designing training sessions, including drills, games and running protocols depicting the characteristics of performance and efforts occurring in real-game situations.

Players with different levels of amputation and lower limb defects need insight. They will likely have a different running performance which needs an adjustment in training and can affect the positional selection criteria. Recognizing and differentiating this early in selecting children with lower limb defects can support self-realization through sport.

Coaches should consider designing training programs to reduce the decrease in the running performance of the team observed in the second half.

The authors see the potential to increase performance in the game efficiency by increasing the changes' frequency. A shorter time spent on the pitch more times, which is in accordance with the amputee football rules, may allow tipping the scales of victory in favour in key moments of the game by maintaining a higher physical disposition for a longer time, especially at the end of the match.

### Limitations of the study

The main limitation is the small number of participants involved in the study. Due to the exclusions of 7 players from the previous 20-man squad, which happened during the study period, finally, there were 13 participants involved for the whole study period, so we could not meet the criteria of 15 participants estimated by the G*Power. Another limitation is the fact that players represented only one team. This limitation is partially offset by the fact that the analyzed matches were against many different opponents, which affected performance and monitored loads.

Although, to the best of the authors' knowledge, this study is by far the longest, considering the two-year monitoring period. It contains the largest number of analyzed matches and is carried out during competitions of the highest sports level. Nevertheless, the readers should be informed and consider the study limitations.

### Further research directions

Further research directions proposed by the authors include repeating the research using a similar methodology in other, larger research groups and comparing GNSS data describing running parameters with the context of events on the pitch and activities during the game. An interesting aspect is also the analysis of the impact of the tactics of changes in relation to the game's intensity and its variability in different periods.

## Conclusions

There are differences between positions in key performance indicators like top speed, peak acceleration, peak deceleration, average distance per minute, GPS load and Gs load, making it possible to create the positional profiles of amputee football players. Players in the midfielder position are the most dynamic—they have the highest top speed, peak acceleration and deceleration, and they run the most distance per minute. Midfielders also have the highest number of impacts and the overall GPS and inertial Gs load per minute. Forwards run the least distance per minute and have the lowest overall GPS load per minute and inertial Gs load per minute. There are no significant differences in number of sprints per minute between forwards, midfielders and defenders. There is a decrease in running performance in the second half of the amputee football match. Despite the observed significant differences between players with different levels of amputation and defect of the lower limb, this effect needs to be further investigated. The trend of higher running performance in lost matches is visible, but this question needs further studies.

## Supplementary Information

Supplementary Information.

## Data Availability

The data presented in this study are available on request from the corresponding author. The data are not publicly available due to good practice in sports because the research was done with players from the National Team, and the number of opponents is very low, which makes it very probable that the opponents could use the data against.
