# Supplementary material for: Match running performance profiles of amputee football players at the national level
Source: Sci Rep. 2023 Jun 19;13:9882. doi: 10.1038/s41598-023-36856-0 (PMC10279752; doi:10.1038/s41598-023-36856-0)
Supplement: Supplementary file 1 — Supplementary Information. [file 41598_2023_36856_MOESM1_ESM.docx]

**Appendix A. Deﬁnitions of Used GNSS Parameters**

A sprint effort begins when an athlete surpasses 4.75(m/s) for at least 1(s). A sprint effort ends when the speed has decreased below 75% of the sprint threshold.

For each sprint, the distance covered is calculated. Sprint total distance is the sum of all distances covered.

For each sprint, the distance covered is calculated. Sprint mean distance is the average of all distances covered.

For each sprint, the duration is calculated. Sprint mean duration is the average of all sprints’ durations.

Inertial Load per minute – Gs (load/min) A scoring value accounting for the intensity and duration of effort based on accelerometer readings. The score is weighted with an exponentially increasing coefﬁcient. Units—arbitrary unit/minute.

GPS Load per minute (load/min) - A scoring value accounting for intensity and duration of efforts based on GPS tracking parameters. The score is weighted with an exponentially increasing coefﬁcient. Units—arbitrary unit/minute.

**Appendix B. GNSS System Technical Details**

Technical parameters of the used GNSS system Titan2 were as follows: GPS ENGINE (U-BloxM8 Concurrent GNSS LCC Module, TCXO, ROM, SAW, LNA); triple GNSS antenna (GPS + GLONASS + GALILEO - SGGP.18A Series Taoglas), high resolution sampling (10HZ), inertial measurement (1000 HZ accelerometer), battery for 7hr, the use of SBAS—EGNOS (Satellite Based Augmentation Stations) (European Geostationary Navigation Overlay Service) error correction.
